# Aging in a highly polluted world: challenges and solutions to prevent Alzheimer’s disease

**DOI:** 10.1007/s00204-026-04469-x

**Published:** 2026-06-08

**Authors:** Jose L. Domingo

**Affiliations:** Universitat Rovira i Virgili, School of Medicine, Laboratory of Toxicology and Environmental Health, Sant Llorens 21, 43201 Reus, Catalonia Spain

**Keywords:** Alzheimer’s disease, Air pollution, Neurodegeneration, Neuroinflammation, Aging, Epigenetics

## Abstract

Alzheimer’s disease (AD) is the most prevalent neurodegenerative disorder globally and a leading cause of disability and death among the elderly. As populations age worldwide, the epidemiological burden of AD is expected to more than double by 2050, surpassing 150 million affected individuals. While genetic susceptibility, particularly the apolipoprotein E ε4 (APOE4) allele, modulates individual risk, most AD cases are late-onset and shaped by complex interactions between genetic background and modifiable environmental exposures. Environmental pollution has emerged as a critical and potentially preventable contributor to this burden. The 2024 Lancet Commission on Dementia Prevention, Intervention, and Care has identified 14 modifiable risk factors, with air pollution explicitly included. Drawing on evidence from human epidemiological cohorts, experimental animal models, and in vitro neuronal/glial systems, the present review aims to synthesize mechanistic evidence linking environmental pollutant classes to AD-relevant neuropathology. The review examines the growing body of evidence linking major categories of environmental pollutants (ambient particulate matter, heavy metals, pesticides, PFAS, and emerging contaminants including microplastics and nanoplastics) to AD risk and pathogenesis. Special attention is given to studies showing that the characteristic neuropathological features of AD may emerge in children and young adults chronically exposed to heavily polluted urban environments, which highlights critical concerns about when and how these changes develop throughout life. Shared mechanistic pathways through which environmental pollutants promote neurodegeneration are discussed, including neuroinflammation, oxidative stress, blood-brain barrier disruption, tau kinase dysregulation, epigenetic reprogramming, and gut-brain axis dysbiosis. The review also examines the amplifying role of biological aging on neurotoxic vulnerability and proposes a comprehensive, multi-level prevention framework addressing individual exposure reduction, clinical risk identification, and population-level policy interventions.

## Introduction

Alzheimer’s disease (AD) is an irreversible, progressive neurodegenerative disorder representing 60–70% of all dementia cases. It is neuropathologically defined by extracellular deposits of amyloid-beta (Aβ) plaques and intraneuronal neurofibrillary tangles (NFTs) composed of hyperphosphorylated tau protein, alongside widespread neuroinflammation, synaptic loss, and neuronal death (DeTure and Dickson 2019). The clinical trajectory encompasses progressive episodic memory failure, executive and language dysfunction, behavioral changes, and eventual loss of independence and life.

The scale of the global AD crisis is unprecedented. In 2024, an estimated 6.9 million Americans aged ≥ 65 years were living with AD dementia, a number projected to reach 13.8 million by 2060 (Alzheimer’s Association, 2024). Globally, at least 55 million people currently live with AD or related dementias, with more than 10 million new cases diagnosed each year, one new case every 3.2 s (Alzheimer’s Disease International, 2024). Unless disease-modifying therapies are developed (Parums 2024), global prevalence is projected to exceed 152 million by 2050 (GBD Dementia Forecasting Collaborators, 2022). These projections reflect not only demographic aging but the complex interplay of genetic susceptibility, lifestyle factors, and environmental exposures.

The 2024 Lancet Commission on Dementia Prevention, Intervention, and Care represents the most comprehensive current synthesis of evidence on modifiable AD risk factors (Livingston et al. 2024). The Commission identified 14 modifiable risk factors, which include -among others- low education, hearing loss, hypertension, physical inactivity, diabetes, depression, smoking, excessive alcohol consumption, social isolation, and critically, air pollution, untreated vision loss, and high LDL cholesterol, collectively accountable for approximately 45% of global dementia cases. This implies that, in principle, almost half of all AD cases could be prevented (or delayed) through targeted interventions, with environmental pollution reduction among the highest-leverage population-level strategies available.

A distinctive and deeply important dimension of the air pollution–AD evidence base is the work conducted by Dr. Calderón-Garcidueñas and co-workers over more than two decades. Studies in Metropolitan Mexico City (MMC), one of the world’s most air-polluted megacities, have reported the neuropathological hallmarks of AD (hyperphosphorylated tau pretangles, amyloid-beta plaques, and inflammatory markers) in forensic autopsy cases of children as young as 11 months and in young adults in their twenties and thirties (Calderón-Garcidueñas et al. 2018, 2024a). The results suggest that, when the cumulative neurotoxic burden of pollutant exposure is sufficiently severe, neuropathological changes consistent with early AD features may be detectable decades before clinical onset, possibly beginning during childhood in heavily polluted environments (Calderón-Garcidueñas 2025). While clinical AD remains overwhelmingly a disease of aging populations, this raises important questions about whether prevention strategies should be extended to earlier life stages in high-exposure populations.

Beyond particulate air pollution, the aging world faces an expanding exposome of other environmental neurotoxicants. The term “exposome” encompasses the totality of environmental exposures across the life course (Wild 2025), shifting focus from single-agent toxicology to the cumulative, interactive nature of real-world exposures. Within it, the term “pollutome” designates the subset of exogenous chemical pollutants (air pollutants, heavy metals, persistent organic pollutants, emerging contaminants), and “neuroactive exposures” the further subset with documented capacity to cross the blood–brain barrier and engage neuroinflammatory, epigenetic, or synaptic mechanisms relevant to AD. These concepts are nested: exposome > pollutome > neuroactive exposures. Ubiquitous heavy metal contamination of food and water, pesticide residues in agricultural environments and food supplies, widespread PFAS contamination of drinking water and consumer products, and the global proliferation of micro- and nanoplastics collectively constitute a pollutome of neuroactive exposures that may cumulatively accelerate AD onset in genetically susceptible individuals (Park et al. 2024). Aging amplifies this vulnerability through multiple biological mechanisms, creating a vicious cycle in which environmental exposures and aging biology reinforce each other’s neurodegenerative effects. It is important to distinguish between association and causation in the current evidence base. Not all substances within a given pollutant category carry equivalent evidence for AD-relevant neurodegeneration. Individual compounds differ substantially in physicochemical properties, biological persistence, and documented mechanisms of neural injury. A critical distinction must also be maintained: neurotoxicity (functional impairment) is not synonymous with neurodegeneration (progressive, irreversible structural loss). While most pollutants reviewed show neurotoxic potential, evidence for causal contribution to AD-type neurodegeneration broadly follows the order: PM2.5 and combustion-derived nanoparticles > lead and cadmium > organochlorine and organophosphate pesticides > arsenic and manganese > PFAS > micro- and nanoplastics, reflecting the convergence of epidemiological, mechanistic, and biomarker evidence elaborated in each section below. While epidemiological and experimental studies increasingly support the contributory role of environmental pollutants in AD pathogenesis, the strength of evidence varies across pollutant classes and study designs. Air pollution, particularly PM_2.5_, is supported by large-scale longitudinal cohorts and meta-analyses, whereas for other exposures such as PFAS and micro- and nanoplastics, the evidence is still emerging and largely derived from experimental or cross-sectional human studies. Consequently, the conclusions drawn in this review should be interpreted within a framework of graded evidence, recognizing that some associations remain provisional and require confirmation through prospective, biomarker-based investigations.

The present review has three main objectives: (1) to comprehensively summarize the epidemiological and mechanistic evidence linking major categories of environmental pollutants to AD risk and pathogenesis, (2) to elucidate the shared biological mechanisms through which diverse pollutants converge on AD-relevant pathology, and (3) to propose a scientifically grounded, multi-level prevention framework. While recent broad-scope syntheses and meta-analyses have effectively catalogued individual pollutant–dementia associations (e.g., Park et al. 2024), they typically do not integrate early-life neuropathological findings or address the critical methodological gaps in real-world mixture toxicology. The present review distinctively links pediatric autopsy evidence with adult-onset epidemiology, critically examines these mixture-exposure limitations, and translates the converging data into a tiered, lifespan-spanning prevention framework explicitly aligned with the 2024 Lancet Commission’s actionable guidelines.

**Fig. 1:**
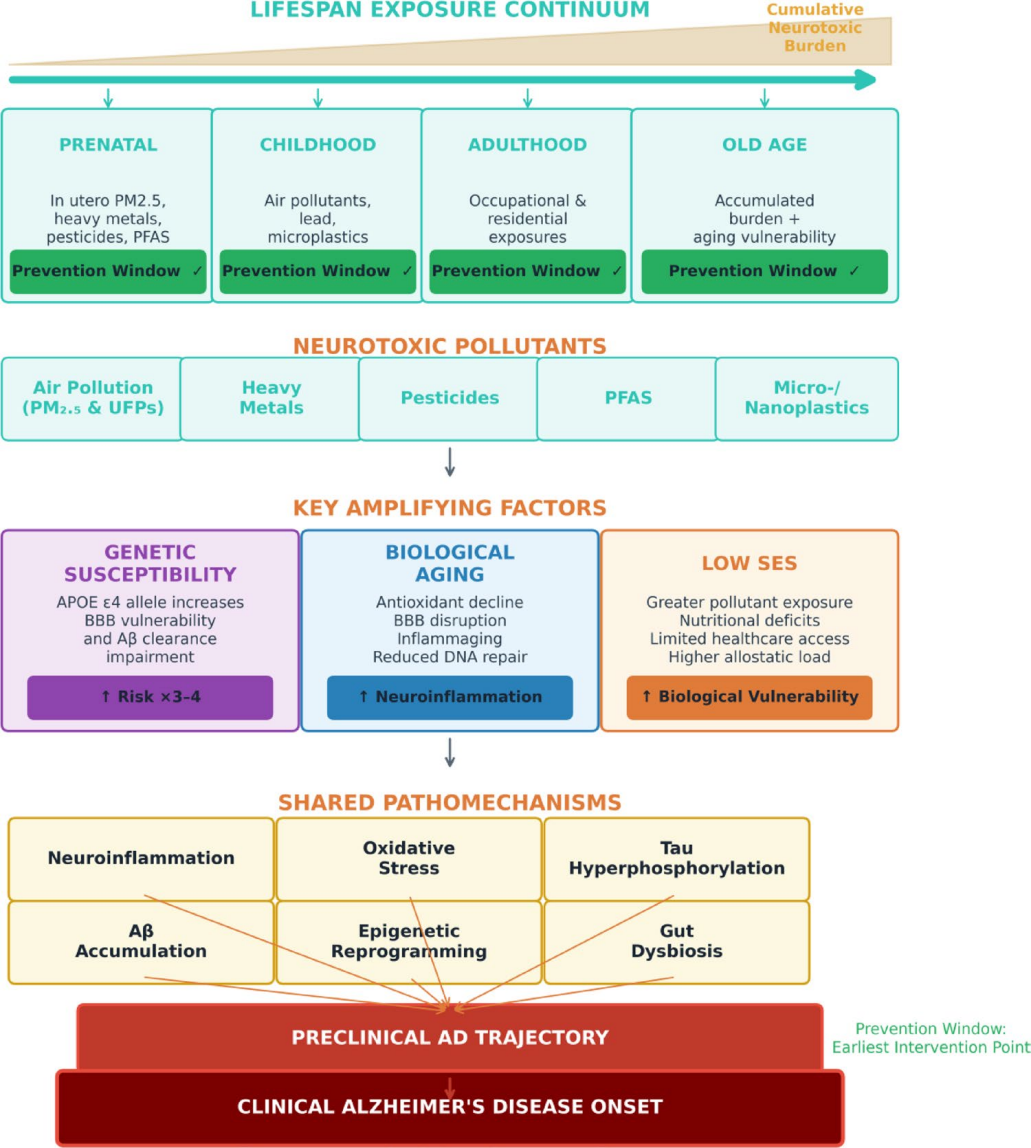
Integrative model linking environmental pollutant exposures to shared pathogenic mechanisms underlying AD, including oxidative stress, neuroinflammation (NF-κB, NLRP3), blood–brain barrier disruption, epigenetic reprogramming, gut–brain axis dysbiosis, and tau kinase dysregulation (GSK-3β, CDK5), collectively promoting amyloid-β accumulation, tau hyperphosphorylation, synaptic loss, and neurodegeneration

To provide an integrative overview of the complex relationships discussed in this review, Fig. 1 links major categories of environmental pollutants with the principal biological mechanisms implicated in AD pathogenesis. The figure illustrates how diverse exposures (ambient particulate matter, heavy metals, pesticides, PFAS, and micro- and nanoplastics) converge on shared pathogenic pathways such as oxidative stress, neuroinflammation, blood–brain barrier dysfunction, epigenetic alterations, and gut–brain axis disruption. These interconnected mechanisms ultimately promote amyloid-β accumulation, tau pathology, synaptic dysfunction, and progressive neurodegeneration. This integrative model highlights the multi-factorial and convergent nature of pollution-related AD risk, providing a framework for interpreting the evidence reviewed in subsequent sections.

## Methods: search strategy

This narrative review was conducted via comprehensive searches of PubMed/MEDLINE, Web of Science and Google Scholar, restricted primarily to articles published between January 2015, and March 2026, with earlier publications included where foundational. Search strategies combined MeSH terms and free-text keywords encompassing: “Alzheimer’s disease,” “AD,” “dementia,” “neurodegeneration,” “air pollution,” “fine particulate matter,” “PM2.5,” “ultrafine particles,” “nanoparticles,” “nitrogen dioxide,” “ozone,” “combustion-derived particles,” “heavy metals,” “lead,” “cadmium,” “arsenic,” “mercury,” “manganese,” “aluminum”, “pesticides,” “organophosphates,” “organochlorines,” “pyrethroids,” “PFAS,” “per-and polyfluoroalkyl substances,” “microplastics,” “nanoplastics,” “neuroinflammation,” “oxidative stress,” “amyloid-beta,” “tau phosphorylation,” “blood-brain barrier,” “gut-brain axis,” “epigenetics,” “cognitive decline,” “environmental health,” “aging,” and “prevention.” Priority was given to systematic reviews, meta-analyses, prospective cohort studies, and original mechanistic research using in vivo or in vitro models with human biomarker validation.

No original data were collected; all findings are derived from a synthesis of peer-reviewed literature. The review does not follow PRISMA methodology and is not registered in PROSPERO, consistent with its narrative scope. The narrative methodology follows the SANRA (Scale for the Assessment of Narrative Review Articles) framework, which does not require a formal search flow diagram but expects transparent reporting of search strategy, inclusion rationale, and critical appraisal (Baethge et al. 2019). Inclusion criteria for this review were: peer-reviewed primary research articles, systematic reviews, and meta-analyses reporting original data or quantitative syntheses on environmental pollutant exposures and AD risk, neuropathology, or mechanistic endpoints. Narrative review articles were included when they provided critical synthesis not covered by primary data. Conference abstracts, preprints, and grey literature were excluded. Studies were selected based on methodological quality (prospective designs preferred for epidemiology, in vivo and human biomarker-validated models preferred for mechanistic studies), relevance to the stated research questions, and publication date (2015–2026, with earlier landmark studies retained). Where systematic reviews or meta-analyses were available, these were preferred over individual studies for epidemiological conclusions.

Although this review follows a narrative approach, particular attention was given to the critical appraisal of study quality and consistency of findings across different methodological designs. Greater weight was assigned to prospective cohort studies, meta-analyses, and investigations incorporating validated clinical or neuropathological endpoints. Experimental studies were evaluated based on biological plausibility, relevance of exposure levels, and translational consistency with human data. Where discrepancies between studies were detected, these were explicitly considered in the interpretation of the evidence.

## Results and discussion

### Global epidemiology and the scale of the AD challenge

The global burden of AD has grown dramatically over the past three decades, driven primarily by aging populations. A Global Burden of Disease (GBD) analysis by Li et al. (2022) reported that the number of individuals living with AD and other dementias was 160.84% higher in 2019 compared to 1990, representing a prevalence of approximately 51.62 million globally. The GBD 2021 Nervous System Disorders Collaborators further reported an accelerating worldwide burden, with dementias constituting a disproportionate share of the neurological disease burden (GBD 2021 Nervous System Disorders Collaborators 2024). Projections from the GBD 2019 Dementia Forecasting Collaborators estimate that the global prevalence will reach 152.8 million by 2050, heavily concentrated in low- and middle-income countries, where environmental pollution exposures are often highest and healthcare resources most constrained (GBD 2019 Dementia Forecasting Collaborators 2022).

The distribution of AD risk is unequal across populations. Among older adults in the United States, Black and Hispanic Americans bear disproportionately high rates of AD, approximately 19% and 14%, respectively, compared with 10% among White older adults (Alzheimer’s Association, 2024). These disparities are due not solely to biological factors, but to differential environmental exposures, which included higher ambient air pollution levels, greater occupational exposures to neurotoxic chemicals, and lower access to preventive healthcare in socioeconomically disadvantaged communities (Josey et al. 2023).

The 2024 Lancet Commission has estimated that 45% of dementia cases worldwide could potentially be prevented by targeting 14 modifiable risk factors, offering a strong rationale for research and policy focused on prevention (Livingston et al. 2024). Environmental pollution (particularly air pollution) is explicitly within this framework, and the Commission notes that improvements in air quality have already contributed to observed declines in age-specific dementia incidence in high-income countries where regulatory action has been taken. To contextualize the relative contribution of pollution, it is instructive to consider the population-attributable fraction (PAF) estimates assigned to each of the 14 Lancet Commission risk factors. Thus, the Commission estimates that air pollution accounts for approximately 9% of the potentially preventable fraction of dementia cases globally, placing it among the higher-impact modifiable factors, behind low education (~ 7% PAF in its own right in high-income settings, but higher globally) and physical inactivity (~ 4% PAF), comparable to or exceeding hypertension and diabetes in terms of attributable burden at the population level in regions with high ambient pollution (Livingston et al. 2024). Interestingly, pollution’s PAF is likely underestimated for low- and middle-income countries, where both exposure levels and the proportion of the population without access to pollution mitigation are substantially higher. Framing pollution reduction within this multi-risk-factor context indicated that no single intervention will eliminate AD risk, but that air quality improvements represent one of the most scalable and equitable strategies available, given their population-level reach and co-benefits for cardiovascular and respiratory health. The major environmental pollutant categories reviewed in the following sections, together with their key epidemiological evidence, quantitative effect estimates, and primary references, are summarized in Table 1.

**Table 1:** Summary of major environmental pollutant categories, key epidemiological evidence, effect estimates, and primary references

Pollutant category	Key epidemiological evidence	Effect estimates	Key references
PM2.5 (Air Pollution)	Multiple large cohort studies (US Medicare, UK Biobank, Three-City Study) and meta-analyses consistently link long-term exposure to increased dementia and AD incidence. Effect found even at levels below current regulatory limits.	HR 1.06 per 3.2 µg/m³ IQR (Shi et al. 2021); +17% dementia risk per 10 µg/m³ (Best Rogowski et al. 2025); +3% per 1 µg/m³ (Abolhasani et al. 2023)	Abolhasani et al. (2023); Best Rogowski et al. (2025); Kim et al. (2025); Shi et al. (2021)
Ultrafine particles and nanoparticles	Forensic autopsy studies in Metropolitan Mexico City (MMC) document AD hallmarks (Aβ plaques, NFTs) in children from 11 months of age. Combustion- and friction-derived magnetic nanoparticles (Fe, Ti, Al) identified in brain tissue and fetal placentas.	AD hallmarks in most MMC residents aged < 40 y; APOE4 carriers have 23.6× higher odds of NFT stage V	Calderón-Garcidueñas et al. (2015, 2018, 2024b, 2025)
Heavy metals (Pb, Cd, As, Hg, Mn)	Epidemiological and mechanistic studies link early-life Pb exposure to late-life AD. Cd crosses BBB and increases Aβ42 and p-tau in hippocampus. As impairs memory via Wnt/β-catenin and NF-κB pathways. Hg disrupts microtubule dynamics.	OR ~ 1.5–3.0 for cognitive impairment at elevated blood Pb levels; dose-response relationship for Cd and As biomarkers (Ahmed et al. 2025; Althobaiti 2025)	Ahmed et al. (2025); Althobaiti (2025); Bihaqi (2019)
Pesticides (OCs, OPs, pyrethroids)	Organochlorines (DDE, dieldrin) elevated in AD brain tissue. OPs inhibit AChE, promote tau hyperphosphorylation, microglial dysregulation, and gut dysbiosis. Case-control and cohort studies show elevated AD risk in agricultural workers.	OR 2–4× for AD among occupationally exposed farmers; CPF accelerates AD in APOE4 transgenic rats (Voorhees et al. 2019; Torres-Sánchez et al. 2023)	Aloizou et al. (2020); Cui et al. (2024); Torres-Sánchez et al. (2023); Voorhees et al. (2019)
PFAS (Per-/Polyfluoroalkyl substances)	Emerging evidence from cerebral organoid models and CNS tissue studies links PFAS to Aβ accumulation, tau phosphorylation, and sphingolipid disruption. Cardiometabolic pathway (hyperlipidemia→atherosclerosis) also implicated. Epidemiological data are currently limited.	PFAS exposure in cerebral organoids induced AD-like neuropathology (Lu et al. 2024); first reported CNS PFAS–AD marker association (Delcourt et al. 2024)	Delcourt et al. (2024); Gardener et al. (2025); Lu et al. (2024)
Micro-/Nanoplastics (MNPs)	MNPs detected in human CSF correlating with AD biomarker profiles. Brain tissue shows highest MNP concentrations of all organs studied, increasing over time. Polystyrene MPs accelerate AD pathology via microglial pyroptosis in animal models.	MNPs in CSF associated with reduced Aβ42 and elevated tau (He et al. 2025); brain MNP levels highest in dementia cases vs. controls (Nihart et al. 2025)	He et al. (2025); Nihart et al. (2025); Wang et al. (2024)

Abbreviations: AD, Alzheimer’s disease; Aβ, amyloid-beta; BBB, blood-brain barrier; HR, hazard ratio; IQR, interquartile range; MNP, micro/nanoplastic; NFT, neurofibrillary tangle; OR, odds ratio; PFAS, per-/polyfluoroalkyl substances; PM, particulate matter.

### Air pollution and AD: epidemiological evidence

Air pollution, particularly fine particulate matter (PM2.5), is the most extensively studied environmental risk factor for AD (Huang et al. 2025; Wilker et al. 2023). PM2.5 refers to airborne particles with an aerodynamic diameter ≤ 2.5 μm generated by combustion processes (motor vehicles, power plants, wildfires, biomass burning), industrial friction, and secondary atmospheric chemistry. PM0.1, or ultrafine particles (UFPs), are even smaller (< 100 nm), and they are increasingly recognized as disproportionately neurotoxic per unit mass due to their deep respiratory penetration, high surface reactivity, and capacity to cross biological barriers directly via the olfactory route (Mussalo et al. 2024; Qin et al. 2024).

The epidemiological evidence linking long-term PM2.5 exposure to AD and dementia is substantial and consistent across study designs, geographic regions, and populations. Shi et al. (2021), analyzing more than 12 million Medicare beneficiaries in the United States from 2000 to 2018, found that each interquartile range (IQR) increase in 5-year average PM2.5 (3.2 µg/m³) was associated with a hazard ratio (HR) of 1.060 (95% CI: 1.054–1.066) for incident dementia and 1.040 (95% CI: 1.032–1.048) for incident AD. A subsequent analysis by the same research group (Shi et al. 2023) extended these findings to the chemical constituents of PM2.5, showing that silicon, vanadium, zinc, and nitrates were independently associated with incident dementia, suggesting pollutant-specific neurotoxic mechanisms beyond particle mass alone.

Meta-analytic syntheses have confirmed and quantified these associations across large multinational datasets. Abolhasani et al. (2023) conducted a systematic review and meta-analysis including 20 cohort studies (total population > 91 million; >5.5 million dementia cases) and reported a 3% increase in dementia risk per 1 µg/m³ increment in PM2.5 (HR 1.03; 95% CI: 1.02–1.05). In turn, Wilker et al. (2023), reporting a meta-analysis published in the British Medical Journal, found an overall HR of 1.04 (95% CI: 0.99–1.09) per 2 µg/m³ PM2.5 increase, rising to 1.42 (95% CI: 1.00–2.02) in studies with active case ascertainment. It is noteworthy that the overall estimate by Wilker et al. (2023) had a confidence interval marginally crossing the null (95% CI: 0.99–1.09), reflecting non-trivial heterogeneity across included studies. This heterogeneity likely reflects differences in study design, exposure assessment methods, case ascertainment strategies, follow-up duration, and geographic variation in pollution composition. The pooled effect estimates from meta-analyses should be interpreted as summary approximations across heterogeneous study populations, rather than as precise point estimates universally applicable to all exposure contexts.

In addition to heterogeneity in exposure assessment and outcome definition, residual confounding is an important consideration in observational studies of air pollution and dementia. Socioeconomic status, education, occupational exposures, access to healthcare, and coexisting cardiovascular risk factors are unevenly distributed across populations and may partially influence observed associations. Although many large cohort studies adjust for these variables, the possibility of residual confounding cannot be fully excluded. This indicates the importance of triangulating evidence across epidemiological, experimental, and neuropathological domains.

Recently, Best Rogowski et al. (2025), analyzed 40 studies including 24 million participants and found that each 5 µg/m³ increase in PM2.5 was associated with an HR of 1.08 (95% CI: 1.02–1.14) for incident dementia, and that black carbon/PM2.5 absorbance showed the strongest association (HR 1.13 per 1 µg/m³). The authors concluded that reducing pollution to WHO-recommended levels could substantially lower population dementia rates. Moreover, a Nature Aging meta-analysis by Huang et al. (2025) applying the Burden of Proof framework across 28 longitudinal cohort studies identified a minimum 14% increased dementia risk averaged across PM2.5 concentrations between 4.5 and 26.9 µg/m³ and found a significant specific association with AD (risk-outcome score indicating moderate-to-strong evidence). At the autopsy-pathology level, Kim et al. (2025), analyzing 602 brain bank cases at the University of Pennsylvania, reported that higher PM2.5 exposure in the year preceding death was significantly associated with greater severity of AD neuropathological change (ADNC), after adjusting for APOE genotype and comorbidities, providing some of the most direct human histopathological evidence available.

Emerging analyses within large Medicare cohorts have also begun to investigate whether the PM2.5–AD association is mediated through cardiovascular and metabolic comorbidities (hypertension, diabetes, depression), or it operates via more direct neurological pathways. While the evidence on mediation remains preliminary, these analyses reinforce the hypothesis that ambient PM2.5 exerts, at least in part, direct neurotoxic effects independent of systemic cardiovascular or metabolic intermediaries. These findings (when/if confirmed) will further strengthen the case for PM2.5 reduction as a brain-protective intervention.

### Ultrafine particles, nanoparticles and AD

Several epidemiological studies focused on PM2.5 (arguably the most alarming evidence regarding air pollution and AD comes from the forensic neuropathological studies) have been conducted in Metropolitan Mexico City (MMC) by Calderón-Garcidueñas and co-workers. MMC is a megacity of 22 million inhabitants chronically exposed to PM2.5 and ozone levels above USEPA standards, with ubiquitous combustion-derived ultrafine particles and industrial nanoparticles in the urban atmosphere. Calderón-Garcidueñas et al. (2018) analyzed 203 forensic autopsies from MMC residents (11 months–40 years), finding tau pretangles in an 11-month-old infant and hyperphosphorylated tau neurites, NFT stages I–II, and amyloid phases 1–2 by adolescence, without other neurological disease. APOE4 carriers had 4.92× higher suicide odds and 23.6× higher odds of reaching NFT stage V than non-carriers with similar age and PM2.5 exposure. Age (*p* = 0.0062) and cumulative PM2.5 (*p* = 0.0178) predicted advanced NFT staging. Combustion-derived, iron-rich, highly oxidative nanoparticles were consistently linked to neurovascular damage. The study concluded that AD beginning in the brainstem of young children affected nearly all young MMC residents examined, representing an urgent public health crisis. This estimate should be interpreted within the specific methodological context of the study population and neuropathological criteria employed. It does not necessarily imply that clinically manifest AD will develop in most exposed individuals. While these findings have substantially advanced the field, their interpretation requires careful consideration. Most of the neuropathological evidence in young individuals derives from a limited number of research groups and specific high-exposure settings. Independent replication in other geographic regions and populations with comparable exposure profiles remains limited. Moreover, the cross-sectional nature of forensic autopsy studies precludes direct inference regarding progression to clinical AD. Therefore, these observations should be interpreted as indicative of early neurobiological alterations potentially related to AD, rather than definitive evidence of established disease.

Recently, Calderón-Garcidueñas et al. (2024b) expanded their analysis using new antibodies and transmission electron microscopy to document overlapping neuropathological hallmarks of AD, Parkinson’s disease, frontotemporal lobar degeneration (FTLD), and amyotrophic lateral sclerosis (ALS) simultaneously in the first two decades of life in the same MMC residents. Brain ultrafine particulate matter and industrial nanoparticles (including various heavy metals) were identified as key mediators of this quadruple neural proteinopathy. Additionally, Calderón-Garcidueñas (2025) published a commentary arguing that air pollution is a major risk factor for the development of AD, cognition deficits, systemic and neural inflammation even in urban children, directly responding to a paper on AD risk factors that insufficiently acknowledged the extensive pediatric evidence. Concurrently, Calderón-Garcidueñas et al. (2025b) reported that cardiovascular damage, arrhythmogenesis, and overlapping AD and Parkinson’s disease pathology begin in pediatric and young adult urban populations through the action of magnetic ultrafine particulate matter and industrial nanoparticles with specific motion behaviors under electromagnetic fields.

A key mechanistic contribution from this research group was the study showing that apparently healthy young urbanites in MMC exposed to PM2.5 above USEPA standards exhibited reduced repressive epigenetic marks (H3K9me2/me3) and increased DNA damage (γ-H2A.X) in frontal white matter neuronal, glial, and endothelial cell nuclei, alongside abundant iron-rich magnetic nanoparticles. This epigenetic reprogramming in young brains exposed to pollution may establish a long-lasting molecular vulnerability that, decades later, manifests as clinical AD (Calderón-Garcidueñas et al. 2020). The research conducted by Calderón-Garcidueñas and colleagues demands a fundamental revision of the preclinical AD concept. AD prevention efforts focused exclusively on adults aged 60 and older are addressing only the final phase of a pathological process that may begin in the nursery. Prevention of pollution-related AD must begin in childhood (or even in utero), given documented nanoparticle presence in fetal brains and placentas (Calderón-Garcidueñas et al. 2022).

**Fig. 2:**
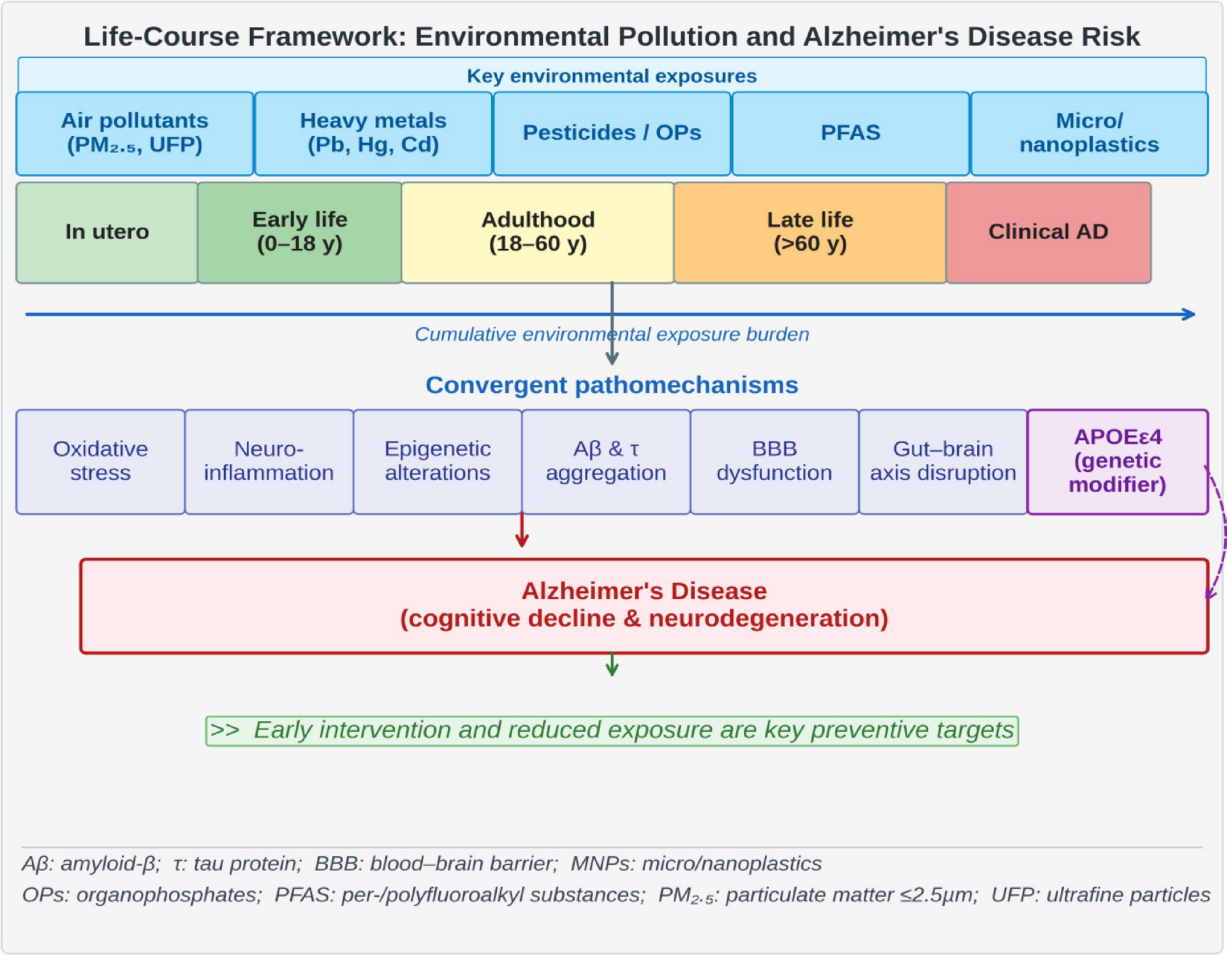
Life-course framework of environmental pollution and Alzheimer’s disease risk

The life-course dimension of pollution-related neurotoxicity is schematically illustrated in Fig. 2. This figure depicts how environmental exposures begin *in utero* and continue throughout childhood, adulthood, and aging, progressively interacting with genetic susceptibility and biological aging processes to influence AD risk. Early life exposure to neurotoxic pollutants may initiate subclinical molecular and cellular alterations, including epigenetic reprogramming and neuroinflammatory priming, which remain latent for decades before manifesting as clinical disease. With advancing age, cumulative exposure burden, declining physiological resilience, and comorbidities amplify these processes, accelerating the transition from preclinical pathology to overt neurodegeneration. This life-course framework remarks the importance of early prevention and supports the need for longitudinal, multi-exposure research approaches.

However, it must be acknowledged that the neuropathological findings reported in children and young adults have not been independently replicated in equivalent forensic autopsy cohorts from other heavily polluted cities. Therefore, certain aspects remain a subject of scientific discussion. Some researchers have questioned whether the hyperphosphorylated tau deposits and amyloid-beta accumulations observed in young urbanites represent incipient true AD’s pathology or, alternatively, pollution-induced neuroinflammatory and proteinopathic changes that are phenotypically similar to, but mechanistically distinct from, the classical AD continuum. The absence of longitudinal follow-up data linking pediatric neuropathological findings to subsequent clinical AD diagnosis, and the difficulty of excluding other confounders in forensic autopsy settings (e.g., socioeconomic factors, nutritional status, concurrent infections), limit the strength of causal inference that can currently be drawn. These considerations do not diminish the substantial public health importance of the work, nor the urgent need for pollution reduction; rather, they remark the value of independent replication and the need for prospective biomarker-based studies in young urban populations exposed to high pollutant burdens.

Recently, Deng et al. (2026) reported the results of a study examining whether hypertension, stroke, and depression mediate or amplify effects of PM_2.5_ on AD in 27.8 million US Medicare beneficiaries. It was found PM_2.5_ exposure significantly increased AD risk, with a slightly stronger association among stroke survivors, while mediation by these comorbidities was minimal (≤ 4.2%). Although PM_2.5_ elevated risks for all three comorbidities, which themselves linked to higher AD risk, the pollution-AD connection operated primarily through direct pathways. It was concluded that stroke might modestly increase susceptibility to air pollution–related cognitive decline. These recent findings highlight air quality improvements as critical for dementia prevention in aging populations, particularly those with overlapping environmental and clinical vulnerabilities.

### Heavy metals and AD

Environmental exposure to neurotoxic heavy metals represents a second major category of pollution-related AD risk (Ahmed et al. 2025; Zaitseva et al. 2025). Lead, cadmium, arsenic, mercury, and manganese are released into the environment through industrial processes, mining, coal combustion, fertilizer use, contaminated water supplies, and food production. The neurotoxic potential and mechanistic pathways of each metal differ substantially by chemical speciation. Lead acts primarily as Pb(II), although organolead compounds are more lipophilic and neurotoxic. Cadmium occurs as Cd(II), with inorganic dietary and waterborne forms representing the main human exposure route. For arsenic, inorganic arsenite As(III) is more toxic than arsenate As(V), with contaminated groundwater as the principal neurotoxicological concern. Mercury speciation is critical. Methylmercury (MeHg), bioaccumulated in fish, crosses the blood–brain barrier far more readily than inorganic forms and is the primary cause of human neurotoxicity. Manganese is most bioavailable as Mn(II), with occupational inhalation posing the greatest neurological risk. These speciation differences are important when interpreting animal studies that typically use single inorganic salts not necessarily reflecting predominant human-relevant exposures.

Lead is among the most thoroughly investigated metals. Bihaqi (2019) reviewed evidence that early-life Pb exposure induces persistent epigenetic changes, including altered DNA methylation at APP and BACE1 regulatory regions. These promote amyloidogenic APP processing and Aβ accumulation decades later, supporting a “developmental origins of AD” hypothesis. A mechanistic study conducted by Liu et al. (2023) demonstrated that chronic Pb exposure increased the affinity of Aβ40 for cerebral vasculature and impaired LRP1 expression in both brain parenchyma and vasculature, facilitating cerebral amyloid accumulation. An epidemiological review focused on elderly women, found that Pb was associated with multiple cognitive impairment biomarkers and that APOE4 carrier status amplified Pb-related cognitive vulnerability (Rashid et al. 2025). In turn, cadmium crosses the BBB and accumulates in the hippocampus, where it triggers oxidative stress via mitogen-activated protein kinase (MAPK) and mTOR pathways, induces neuroinflammation, and elevates hippocampal Aβ42 and phosphorylated tau levels in animal models (Arruebarrena et al. 2023). A mechanistic review by Ahmed et al. (2025) found that while Pb, Cd, As, and Mn shared mechanistic pathways, tau phosphorylation, mitochondrial dysfunction, altered Aβ processing, oxidative stress, and autophagy impairment, certain effects are unique to specific metals, emphasizing the importance of studying these exposures individually and in mixtures.

On the other hand, arsenic, ingested primarily through contaminated drinking water in many low- and middle-income countries, has been shown to impair spatial learning and memory in rodent models, activate NF-κB inflammatory signaling, alter tau kinase activity (CDK5), and disrupt the Wnt/β-catenin pathway through Akt inhibition (Hua et al. 2024). Althobaiti (2025) examined heavy metal-AD therapeutic approaches, concluding that chelation therapy (for Pb, Cd, Hg) and antioxidant supplementation (N-acetylcysteine, alpha-lipoic acid) are the most mechanistically plausible adjunctive interventions, while emphasizing that primary prevention through exposure reduction remains the most effective strategy. In turn, mercury, particularly in its organic methylmercury form derived mainly from fish consumption and inorganic forms from occupational or dental amalgam sources, has been linked to AD through microtubule destabilization (relevant to tau pathology), oxidative stress, and mitochondrial dysfunction. Islam et al. (2022) reviewed neurotoxicity of multiple metals, noting that Hg promotes misfolding of tau protein at concentrations achievable in chronically exposed individuals. The importance of an epigenetic mechanism was highlighted by Venkatesan et al. (2024), who reviewed how Cd, Fe, As, Cu, and Li exert epigenetic effects (histone modification and alteration of DNA methylation at AD-relevant gene loci), which could accelerate disease progression.

Overall, although the evidence linking heavy metal exposure to AD spans mechanistic, experimental, and epidemiological domains, it remains heterogeneous in strength across individual metals. For lead and cadmium, converging experimental and human data provide moderate support for a contributory role in neurodegeneration, whereas for other metals the evidence is more variable and often limited to mechanistic studies. Future research integrating longitudinal exposure assessment with validated cognitive and biomarker outcomes should be essential to clarify causal relationships.

A critical appraisal of this evidence reveals important limitations. Most human studies are cross-sectional and rely on a single biomarker measurement, inadequately capturing cumulative lifetime exposure. Confounders such as socioeconomic status, smoking, dietary patterns, and co-exposure to other neurotoxicants are inconsistently controlled. Moreover, most experimental studies use exposure levels that substantially exceed realistic human environmental exposures, limiting their translational relevance. Stronger evidence from prospective, biomarker-validated studies with rigorous confounder adjustment is needed before causal conclusions can be drawn (Ahmed et al. 2025; Althobaiti 2025).

### Aluminum and AD: a potential neurotoxic link

Aluminum (Al) is the most abundant metallic element in the Earth’s crust and a recognized environmental neurotoxin with no known biological function. Human body burden of Al has increased in recent decades due to its widespread use in food processing, packaging, drinking water treatment, and the acidification of water resources by acid rain (Hiller et al. 2023; Yokel 2025). Aluminum crosses the BBB, increases oxidative stress and lipid peroxidation, generates ROS, disrupts iron homeostasis, and activates inflammatory and apoptotic pathways, thereby promoting amyloid-β (Aβ) accumulation and tau hyperphosphorylation, the two hallmark pathological features of Alzheimer’s disease (AD) (Colomina and Peris-Sampedro 2017). Neuroimaging studies have revealed gray matter volume reduction in the hippocampus and frontal lobes of aluminum-exposed individuals, accompanied by disrupted functional connectivity and diminished white matter integrity, changes that may synergistically interact with Aβ aggregation and neurofibrillary tangle formation (Hu et al. 2026). At the epidemiological level, a recent systematic review and meta-analysis (synthesizing 54 eligible studies) confirmed a consistent association between environmental Al exposure and the risk of AD across diverse study designs and geographic contexts (Soleimani et al. 2025). Nonetheless, causality remains debated: while Al causes tau and Aβ accumulation in experimental animals and induces neuronal apoptosis in vivo and in vitro, some individuals chronically exposed to Al through water and food do not develop AD pathology, apparently because of a more effective gastrointestinal barrier (Kandimalla et al. 2016). Overall, current evidence supports Al as a plausible environmental contributor to AD pathogenesis, although its etiological weight relative to genetic and other risk factors requires further clarification.

### Pesticides and AD

Pesticides are a chemically diverse group of more than 1,000 active compounds used globally in agriculture, public health, and domestic pest control. Several classes have been implicated in AD risk, with the strongest evidence for organochlorines (OCs), organophosphates (OPs), and, to a lesser extent, pyrethroids, carbamates, and neonicotinoids. Within the organochlorine class, p,p’-DDE and dieldrin have been most consistently detected at elevated concentrations in post-mortem AD brain tissue. Among organophosphates, chlorpyrifos is the most extensively studied, with data from epidemiological cohorts of older adults and transgenic AD rodent models. For pyrethroids and carbamates, evidence remains largely experimental, and neonicotinoid data are restricted to early-stage cellular studies. The most direct human evidence links organochlorines to AD in adults aged > 60 years. In turn, the organophosphate–AD association derives primarily from occupationally exposed agricultural workers, while mechanistic endpoints (tau hyperphosphorylation, AChE inhibition, microglial activation) come predominantly from in vitro and rodent models. Organochlorine compounds such as DDT/DDE, aldrin, dieldrin, and lindane are persistent organic pollutants (POPs) that bioaccumulate in adipose tissue, being detectable in human blood decades after their agricultural prohibition. Multiple case-control and epidemiological studies have reported high brain or blood concentrations of OC compounds in AD patients compared to controls, with some demonstrating dose-response relationships (Aloizou et al. 2020). Dieldrin in particular, has been found at high concentrations in post-mortem AD brain tissue in several studies, whereas rodent studies link dieldrin to mitochondrial dysfunction and dopaminergic neurodegeneration (Alehashem et al. 2024).

Organophosphate pesticides are mechanistically highly relevant given that their primary mode of action (irreversible acetylcholinesterase (AChE) inhibition) directly disrupts the cholinergic system, which is centrally impaired in AD. Voorhees et al. (2019) showed that occupational-like chlorpyrifos (CPF) exposure in the TgF344-AD transgenic rat model caused chronic microglial dysregulation and significantly accelerated AD-like neurodegeneration and cognitive deficits, particularly in males. It supports the “two-hit” hypothesis in which environmental OP exposure provides a pathological amplifying signal in genetically predisposed individuals. Recently, Yadav et al. (2024) reviewed OP-induced inflammatory responses in microglia and astrocytes, showing how CPF-like exposures trigger NF-κB signaling, reactive gliosis, mitochondrial dysfunction, and progressive neuroinflammation contributing to AD pathogenesis.

Torres-Sánchez et al. (2023) reviewed the effects of OC, OP, carbamate, pyrethroid, and neonicotinoid pesticides on tau phosphorylation. All five classes were found to alter the enzymatic balance between glycogen synthase kinase-3 beta (GSK-3β) and protein phosphatase-2 A (PP2A), shifting the balance toward tau hyperphosphorylation at multiple AD-relevant sites including Ser202, Thr205, Ser396, and Ser404, and to promote neuroinflammation through microglial activation. On the other hand, the gut-brain axis has emerged as an additional mechanistic pathway. Cui et al. (2024) reported that the OP insecticide malathion, at environmentally relevant concentrations, induced AD-like cognitive impairment and Aβ accumulation in both wild-type and APP/PS1 transgenic mice through gut microbiota dysbiosis, depleting beneficial Lactobacillus and Akkermansia while increasing Dubosiella. This dysbiosis led to gut barrier impairment, tryptophan/kynurenine pathway disruption, and activation of the aryl hydrocarbon receptor (AhR), creating a neuroinflammatory milieu that promoted AD-like pathology. In turn, Kiani et al. (2023) examined case-control data on pesticide exposure and AD, finding that participants with documented organophosphate or organochlorine exposure had significantly elevated oxidative stress biomarkers compared to unexposed controls, consistent with oxidative stress as a central mechanism of pesticide-related AD risk.

The epidemiological evidence linking pesticides to AD requires critical evaluation. Most human studies are designed as case-control, introducing recall bias and limiting causal inference. Sample sizes are frequently modest, and key confounders (co-exposure to other agrochemicals, nutritional status, and occupational physical activity) are inconsistently reported. Objective biomarkers of pesticide exposure are lacking in many studies. Furthermore, experimental evidence is predominantly derived from transgenic rodent models using individual compounds, whereas real-world human exposures involve complex mixtures whose combined effects remain largely unstudied (Aloizou et al. 2020; Domingo 2026; Torres-Sánchez et al. 2023).

### PFAS and AD

Per-and polyfluoroalkyl substances (PFAS) comprise hundreds of synthetic fluorinated compounds that are exceptionally resistant to environmental and biological degradation, justifying the designation “forever chemicals.” PFAS are found in non-stick cookware, food packaging, water-resistant textiles, firefighting foams, personal care products, and many other consumer items (Glüge et al. 2020; Yang et al. 2025). The USEPA’s April 2024 National Primary Drinking Water Regulation set the first legally enforceable maximum contaminant levels (MCLs) for six PFAS, reflecting growing concern about their public health impact (Gardener et al. 2025).

Unlike lipophilic POPs, PFAS bind to proteins and accumulate in protein-rich organs including the liver, kidneys, bone marrow, and brain (Domingo 2025). Their neurotoxic and cardiometabolic effects are well-documented (Gardener et al. 2025; Schillemans et al. 2024). Mechanistically, PFAS can disrupt thyroid hormone signaling (important for brain development and maintenance), induce neuroinflammation, impair BBB integrity, and promote hyperlipidemia, which is itself a dementia risk factor (Gardener et al. 2025). Lu et al. (2024) reported that cerebral organoids derived from human induced pluripotent stem cells (iPSCs) exposed to a PFOA, PFOS, and PFHxS mixture at environmentally relevant concentrations over 35–70 days exhibited Aβ accumulation and tau hyperphosphorylation characteristic of AD neuropathology. Lipidomic analyses revealed significant disruption of sphingolipid metabolism, suggesting a specific metabolic pathway through which PFAS promotes AD-like changes. In turn, Delcourt et al. (2024) found the first observational evidence of an association between PFAS accumulation in human CNS tissue and markers of AD, providing translational support for the experimental data.

In a recent review, Gardener et al. (2025) assessed the current evidence base and proposed a new longitudinal study, the PFAS VascCog Longitudinal Study within the Northern Manhattan Study cohort, to prospectively examine 13 serum PFAS concentrations in relation to cognitive outcomes. The authors hypothesized a mechanistic pathway through which PFAS-induced hyperlipidemia and atherosclerosis mediate dementia risk, in addition to direct neurotoxic effects. It was found that while epidemiological data on PFAS-dementia associations remain limited, the convergence of neurotoxicological, experimental, and biomonitoring evidence warrants urgent prioritization of research in this area.

It must be noted that the epidemiological evidence specifically linking PFAS to AD remains sparse and methodologically heterogeneous. The existing human data derive from cross-sectional or small observational studies, while the analytical techniques for quantifying PFAS in brain tissue are still being validated. Consequently, the conclusions regarding PFAS and AD should be considered hypothesis-generating rather than conclusive, and the certainty assigned to PFAS in the current review is necessarily lower than that for PM2.5 or heavy metals, where large prospective cohort data and multiple independent meta-analyses are available. In fact, the weight of evidence does not allow PFAS to be considered established risk factors for AD, but rather as plausible contributors requiring investigations within well-designed longitudinal studies.

A further concern is that existing studies rarely account for the full range of potential confounders, including cardiometabolic comorbidities, dietary patterns, and co-exposure to other environmental chemicals, all of which may correlate with both PFAS exposure and dementia risk. The experimental cerebral organoid data, while mechanistically informative, cannot be directly extrapolated to the chronic, low-level, multi-compound PFAS exposures typical of the general human population (Gardener et al. 2025; Lu et al. 2024). Until large prospective studies with comprehensive confounder adjustment are available, the association between PFAS and AD must be considered biologically plausible but epidemiologically unconfirmed.

### Micro- and nanoplastics and AD

Micro- and nanoplastics (MNPs) represent the most recently recognized category of ubiquitous environmental contaminants with potential neurotoxic relevance (Gecegelen et al. 2025; Sofield et al. 2024). Microplastics (MPs, < 5 mm) and nanoplastics (NPs, < 1 μm) are generated by the fragmentation and degradation of larger plastic debris, by industrial manufacturing of nano-sized polymers, and by abrasion of synthetic textiles. Adults are estimated to ingest up to 121,000 MPs annually through food, drinking water, and inhalation (Dennis et al. 2025; Ziani et al. 2023). The global plastic production rate ensures that environmental MNP concentrations will continue to rise for decades.

Evidence of MNP penetration into the human CNS has been directly reported. Nihart et al. (2025) compared MP accumulation in human kidney, liver, and brain tissue samples collected between 2016 and 2024. It was found that brain tissues had the highest MP concentrations of all organs studied, with levels increasing over time. Most importantly, the 2025 analysis found significant MP accumulation specifically in glial cells and cerebrovascular walls of individuals with documented dementia, providing direct histological evidence linking CNS microplastic burden to neurodegeneration (Nihart et al. 2025).

Recently, He et al. (2025) detected MPs in human cerebrospinal fluid (CSF). It was observed that their presence correlated inversely with the CSF Aβ42/40 ratio and positively with p-tau, AD’s most established CSF biomarker signature, suggesting that CNS microplastic exposure might be associated with ongoing AD neuropathological processes. Despite these findings, caution is warranted in interpreting the current human data. The available studies are limited by small sample sizes, potential contamination during sample processing, and the absence of standardized analytical methodologies. Consequently, while the detection of MPs in human brain and cerebrospinal fluid is of considerable interest, its direct relevance to AD pathogenesis remains to be firmly established.

Wang et al. (2024) demonstrated experimentally that polystyrene MP exposure accelerated cognitive impairment progression in 5xFAD transgenic AD mice through induction of microglial pyroptosis, an inflammatory programmed cell death mediated by the NLRP3 inflammasome, providing a cellular mechanism for MNP-driven neuroinflammation. In turn, Gecegelen et al. (2025) assessed the emerging evidence on MNPs as a novel dementia risk factor, identifying BBB disruption, mitochondrial dysfunction, oxidative stress, and microglial activation as key pathological mechanisms. The authors highlighted that nano-sized plastics with a biomolecular corona of cholesterol and phospholipids show enhanced BBB penetration, and called for urgent longitudinal human epidemiological studies and standardization of MNP quantification methods in biological matrices. Collectively, the evidence on MNPs and AD must be considered as preliminary. Human epidemiological investigation is currently limited to a small number of cross-sectional studies with modest sample sizes. Thus, causal inference from these data is premature. The mechanistic evidence is predominantly derived from animal models and in vitro systems using polystyrene particles at concentrations that could not fully reflect real-world human exposures. Furthermore, the absence of standardized analytical methods for MNP quantification in brain tissue and cerebrospinal fluid, impedes inter-study comparisons. Future well-designed prospective cohort studies incorporating validated MNP biomarkers will be essential to establish whether the associations found to date reflect genuine causal neurotoxicity.

Potential confounding factors also warrant careful evaluation. People with elevated MNP concentrations often exhibit dietary or lifestyle characteristics that are independently linked to dementia risk, a relationship, which has not yet been adequately controlled in existing human research. Moreover, the possibility of reverse causation cannot be ruled out (neuroinflammation and impaired glymphatic function associated with dementia might promote MNP accumulation rather than result from it). Finally, measuring MNPs in biological samples remains technically challenging and susceptible to contamination artifacts unless standardized analytical procedures are applied (Gecegelen et al. 2025; Nihart et al. 2025). Taken together, these points highlight the importance of interpreting current evidence with caution.

### Shared pathomechanisms: convergence of environmental pollutants on AD pathology

A key conceptual advance emerging from recent research is the recognition that diverse environmental pollutants do not act through isolated mechanisms but rather converge on a limited number of interconnected biological pathways. This convergence suggests that AD risk may be more accurately understood within an exposome framework, in which cumulative and interacting exposures across the lifespan shape disease susceptibility. Such approach provides a unifying perspective that integrates chemical diversity with mechanistic commonality. Despite the chemical diversity of the pollutants discussed above, a convergent set of biological mechanisms mediates their neurotoxic and pro-AD effects (Table 2). Understanding these shared pathways is essential for developing broadly applicable preventive and therapeutic strategies. Key genes implicated in each signaling pathway are identified in the subsections below and incorporated into Table 2: *NFKB1/RELA* (NF-κB neuroinflammatory signaling), *NLRP3/CASP1* (inflammasome assembly), *BACE1/APP/PSEN1/PSEN2* (amyloidogenic processing), *MAPT* (tau), *GSK3B/CDK5* (tau kinase dysregulation), *CLDN5/OCLN/TJP1* (blood–brain barrier integrity), *NFE2L2/SOD1/SOD2/GPX1* (antioxidant defense), *DNMT3A/HDAC2* (epigenetic regulation), *IDO1/AHR/TLR4* (gut–brain axis signaling), *BDNF/NTRK2* (synaptic resilience), and *APOE* (lipid transport and amyloid clearance).

**Table 2:** Key biological mechanisms linking environmental pollutants to Alzheimer’s disease pathology

Mechanism	AD pathological outcome	Key pollutants involved	Experimental evidence
Oxidative stress / ROS generation	Tau hyperphosphorylation (GSK-3β/CDK5 activation); amyloidogenic APP processing (BACE1 upregulation); mitochondrial dysfunction	PM2.5, PM0.1, Pb, Cd, As, Hg, organophosphates, PFAS, polystyrene MNPs	Reduced glutathione and SOD in metal-exposed rodents; ROS-mediated BACE1 activation in PM2.5-exposed neuronal cells (Cristaldi et al. 2022; Roy et al. 2023)
Neuroinflammation	Microglial/astrocyte activation; TNF-α, IL-1β, IL-6 overproduction; impaired Aβ clearance; synaptic damage; complement activation	PM2.5, UFPM (nanoparticles), Pb, Cd, OPs, PFAS, polystyrene MNPs, nanoplastics	COX-2/IL-1β upregulation in frontal cortex of MMC children (Calderón-Garcidueñas et al. 2008); microglial pyroptosis with polystyrene MPs (Wang et al. 2024)
Blood-Brain Barrier (BBB) disruption	Increased CNS entry of neurotoxicants; peripheral inflammatory mediator influx; impaired Aβ clearance via LRP1; cerebral amyloid angiopathy	PM2.5, UFPM, Pb, Cd, PFAS, nanoplastics	Pb reduces LRP1 expression in brain vasculature (Liu et al. 2023); PFAS impairs BBB tight junctions in cell models; nanoparticles in cerebrovascular walls of dementia cases (Nihart et al. 2025)
Epigenetic reprogramming	Altered DNA methylation of APP, BACE1, APOE; histone H3K9me2/me3 reduction; non-coding RNA dysregulation; long-lasting gene expression changes	Pb, Cd, As, PM2.5 (epigenetic effects documented); OPs	Reduced H3K9me2/me3 in frontal white matter of MMC residents exposed to PM2.5 (Calderón-Garcidueñas et al. 2020); Pb-induced DNA methylation changes at BACE1 locus (Bihaqi 2019)
Gut-brain axis dysbiosis	Reduced beneficial bacteria (Lactobacillus, Akkermansia); increased gut permeability; decreased SCFA production; systemic endotoxemia promoting neuroinflammation	OPs (malathion, chlorpyrifos), PFAS, MNPs, PM2.5 (ingested fraction)	Malathion-induced gut dysbiosis with tryptophan/kynurenine pathway disruption and AD-like pathology (Cui et al. 2024); MNPs alter gut microbiome composition in rodent models
Tau kinase dysregulation	Tau hyperphosphorylation at Ser198/202, Thr205, Ser396/404; neurofibrillary tangle formation; impaired microtubule stability	OCs, OPs, carbamates, pyrethroids, neonicotinoids; As, Pb	All five pesticide classes alter GSK-3β/PP2A enzymatic balance driving tau hyperphosphorylation (Torres-Sánchez et al. 2023); As activates CDK5 in neuronal cells
RNA epitranscriptomic modification	Altered m6A RNA methylation; upregulation of PTGS2 (prostaglandin synthesis); disrupted transcript stability, splicing, and translation	PM2.5 (documented); likely other oxidative pollutants	PM2.5 increases m6A modifications and PTGS2 expression in brain-relevant cell models (Li et al. 2023; Park et al. 2024)

Abbreviations: Aβ, amyloid-beta; BBB, blood-brain barrier; CDK5, cyclin-dependent kinase 5; GFAP, glial fibrillary acidic protein; GSK-3β, glycogen synthase kinase-3 beta; LPS, lipopolysaccharide; MAPK, mitogen-activated protein kinase; MNP, micro/nanoplastic; mTOR, mechanistic target of rapamycin; NF-κB, nuclear factor kappa B; NLRP3, nucleotide-binding oligomerization domain-like receptor protein 3; OC, organochlorine; OP, organophosphate; PFAS, per-/polyfluoroalkyl substances; PP2A, protein phosphatase 2 A; ROS, reactive oxygen species; SCFA, short-chain fatty acid; SOD, superoxide dismutase; UFPM, ultrafine particulate matter.

Oxidative stress is the most consistently reported mechanism across all pollutant categories. Fine and ultrafine particulate matter (PM2.5 and PM0.1), heavy metals (lead, cadmium, arsenic, and mercury), organophosphate pesticides, PFAS, and MNPs, all generate reactive oxygen species (ROS) and reactive nitrogen species (RNS), which overwhelm cellular antioxidant defense systems, leading to cumulative oxidative damage. The resulting oxidative damage to lipids, proteins, and nucleic acids is particularly injurious to neurons, which have high metabolic rates, limited antioxidant capacity, and minimal regenerative potential. Oxidative stress activates GSK-3β and CDK5, promoting tau hyperphosphorylation, and upregulates BACE1 via NF-κB and other redox-sensitive transcription factors, shifting APP processing toward the amyloidogenic pathway and increasing Aβ production (Cristaldi et al. 2022; Roy et al. 2023).

Neuroinflammation is a second universal mechanism. Microglial and astrocyte activation by environmental pollutants leads to sustained release of TNF-α, IL-1β, IL-6, COX-2, and complement proteins, establishing a chronic neuroinflammatory state that promotes tau pathology, synaptic damage, and neuronal apoptosis (Block and Calderón-Garcidueñas 2009; Heneka et al. 2015). Calderón-Garcidueñas et al. (2008) reported COX-2/IL-1β upregulation in the olfactory bulb, frontal cortex, substantia nigra, and vagus nerve of young MMC urbanites alongside early Aβ42 and α-synuclein immunoreactivity, demonstrating that neuroinflammation in air-polluted environments was measurable and progressive from childhood. Wang et al. (2024) showed that microplastic-induced microglial pyroptosis via the NLRP3 inflammasome represents a newly recognized inflammatory pathway relevant to AD.

Blood-brain barrier disruption is a critical enabling node. Environmental pollutants, including PM2.5, Pb, Cd, PFAS, and MNPs, impair BBB integrity through endothelial oxidative stress, tight junction protein (claudin-5, occludin, ZO-1) degradation, and pericyte loss (Hussain et al. 2023). Disruption of the BBB allows entry of peripheral inflammatory mediators, circulating Aβ, and neurotoxic ions into the CNS, amplifying neuroinflammatory cascades and facilitating Aβ accumulation. Calderón-Garcidueñas et al. (2020) found neurovascular unit damage in young MMC residents, with NPs identified within endothelial cell nuclei.

Epigenetic reprogramming represents a mechanism of particular importance for the link between early-life exposures and decades-later AD onset. Pb, Cd, As, and PM2.5 are well-documented epigenetic toxicants. Calderón-Garcidueñas et al. (2020) found reduced H3K9me2/me3 (repressive histone methylation marks) and increased DNA double-strand breaks (γ-H2A.X) in young MMC residents’ brain tissue, consistent with widespread chromatin decompaction and genomic instability that could alter expression of AD-relevant genes including APP, BACE1, and tau. In turn, Bihaqi (2019) reviewed Pb-induced persistent methylation changes at BACE1 regulatory sequences that promote amyloidogenic processing into old age, while Torres-Sánchez et al. (2023) showed that pesticide-induced imbalances in GSK-3β/PP2A promote not only tau hyperphosphorylation but also neuroinflammatory gene expression through NF-κB.

Gut-brain axis dysbiosis is an emerging mechanistic pathway through which oral pollutant ingestion may promote CNS neuroinflammation. Environmental pollutants ingested orally disrupt the gut–brain axis through several converging mechanisms: depletion of SCFA-producing commensals (*Lactobacillus* spp., *Akkermansia muciniphila*, *Faecalibacterium prausnitzii*) that maintain intestinal barrier integrity and suppress neuroinflammation via GPR41/GPR43. Also, expansion of LPS-producing Proteobacteria causing metabolic endotoxemia, shift of tryptophan metabolism toward the kynurenine pathway (quinolinic acid, an NMDA agonist), AhR upregulation amplifying inflammatory signaling, and tight junction degradation (*OCLN*, *CLDN1*, *IDO1*, *NLRP3*) increasing intestinal permeability and LPS translocation, triggering microglial priming via TLR4/NF-κB. Gut-derived signals reach the brain via the vagus nerve and circulating cytokines (IL-6, TNF-α, IL-1β), promoting tau hyperphosphorylation and amyloidogenic APP processing. Cui et al. (2024) reported that malathion-induced gut dysbiosis, with depletion of Lactobacillus and Akkermansia and activation of the colonic aryl hydrocarbon receptor (AhR) via kynurenine pathway, drove neuroinflammation and AD-like pathology. Similarly, PFAS and MNPs have been shown in rodent models to alter gut microbial community composition, reduce short-chain fatty acid (SCFA) production, and increase intestinal permeability, facilitating the translocation of endotoxins (LPS) to the systemic circulation and ultimately promoting microglial priming in the brain (Sofield et al. 2024).

RNA epitranscriptomic modification via N6-methyladenosine (m6A) represents a recently discovered mechanism linking PM2.5 to AD-relevant transcriptional changes. Li et al. (2023), demonstrated that PM2.5 exposure is associated with increased m6A modifications in the brain and upregulation of prostaglandin-endoperoxide synthase 2 (PTGS2), a key inflammatory enzyme, through stabilization of its transcript. This pathway connects air pollution exposure to sustained neuroinflammatory gene expression in a potentially persistent epigenetic manner. A structured summary of the seven major mechanistic pathways through which environmental pollutants promote AD pathology, the AD-relevant outcomes they drive, the pollutants implicated in each, and the experimental evidence supporting each pathway is shown in Table 2.

### Aging as an amplifier of pollutant neurotoxicity

The exponential increase in AD prevalence with age, 5% for ages 65–74, 13.2% for ages 75–84, and 33.4% for those ≥ 85 years (Alzheimer’s Association, 2024), reflects not simply the cumulative burden of pathological processes but also progressive deterioration of the biological systems that normally buffer against neurotoxic insults. Aging creates a state of heightened vulnerability to environmental pollutants through multiple converging mechanisms. The transition from pollutant-induced neurotoxicity to irreversible neurodegeneration in the aging brain involves five converging mechanisms: (a) chronic microglial priming via NF-κB, NLRP3, and complement activation, sustaining neurotoxic levels of TNF-α, IL-1β, and IL-6, (b) accumulation of misfolded Aβ and hyperphosphorylated tau that overwhelms declining proteasomal and autophagic clearance (*BECN1*, *ATG5*, *TFEB*), (c) mitochondrial dysfunction driven by pollutant-induced ROS, impairing ATP production and activating intrinsic apoptosis (*BAX*, *CASP3*), (d) epigenetic reprogramming at *APP*, *MAPT*, *PSEN1/2*, *APOE*, and *BACE1* loci that shifts APP processing toward the amyloidogenic pathway, and (e) progressive loss of synaptic resilience through downregulation of BDNF/TrkB, *CAMKII*, and PSD-95, converting reversible synaptic dysfunction into permanent synapse loss. Elucidating these transitions requires longitudinal biomarker-based studies in human populations with documented lifetime exposure profiles. First, the aging brain experiences progressive decline in antioxidant enzyme activity (superoxide dismutase (SOD), catalase, and glutathione peroxidase) reducing its capacity to neutralize the ROS generated by pollutant exposures (Yu et al. 2025). This age-related oxidative vulnerability is further compounded by accumulating mitochondrial DNA mutations and declining mitochondrial quality control, both of which amplify the pro-oxidant impact of environmental toxicants. Second, age-related impairment of autophagy and proteasomal degradation reduces clearance of misfolded proteins, allowing Aβ oligomers and tau aggregates to accumulate in the face of pollutant-induced overproduction (Gao et al. 2025). Third, the aging BBB becomes progressively more permeable due to pericyte loss, endothelial dysfunction, and tight junction deterioration, facilitating greater entry of circulating neurotoxicants into the CNS (Knox et al. 2022). Fourth, the phenomenon of inflammaging (chronic, sterile, low-grade systemic inflammation characteristic of biological aging) creates a permissive neuroinflammatory environment in which pollutant-induced microglial activation can more easily exceed pathological thresholds and sustain chronic neuroinflammation (Tamatta et al. 2025). Fifth, age-related decline in lymphatic and glymphatic CNS waste clearance impairs the normal overnight flushing of Aβ and other toxic proteins from the brain, amplifying the impact of pollutant-induced production increases (Xiong et al. 2024). Sixth, APOE4 genotype, the strongest genetic risk factor for late-onset AD, further modulates the aging-pollution interaction by impairing Aβ clearance via lipoprotein receptor pathways and by increasing the brain’s sensitivity to oxidative and inflammatory stress, which has been extensively shown in the MMC cohorts by Calderón-Garcidueñas et al. (2017, 2018, 2023).

The synergy between aging biology and environmental pollution creates a vicious cycle: pollutants accelerate neurodegeneration, while neurodegeneration impairs the cellular resilience that would otherwise moderate pollutant toxicity. This cycle is amplified by the genomic instability, telomere shortening, and mitochondrial dysfunction that characterize the aging nervous system. Understanding this synergy underscores why the oldest and most biologically frail individuals suffer the greatest consequence from pollution exposures, while simultaneously highlighting that reducing early-life and midlife pollution burdens can meaningfully alter the long-term trajectory toward AD (Kareem et al. 2025; Malecki et al. 2022).

### Prevention strategies at individual, clinical, and population levels

Given the evidence that multiple environmental pollutants contribute substantially to AD risk and that aging amplifies this vulnerability, a coordinated, multi-level prevention framework is required. Such a framework must address exposures across the lifespan and operate simultaneously at individual, clinical, and policy levels. Table 3 provides a structured summary of evidence-based prevention strategies organized by intervention level.

**Table 3:** Multi-level prevention strategies for pollution-related Alzheimer’s disease risk

Level of intervention	Target/action	Rationale	Key evidence
Individual—exposure reduction	Use HEPA air purifiers indoors; choose low-traffic routes; avoid biomass burning; use filtered water; choose organic produce and low-mercury fish; avoid PFAS-containing cookware and packaging	Reduces personal PM2.5, PFAS, heavy metal, and pesticide body burden; directly addresses the most prevalent exposure pathways	Wang et al. (2022) showed improved air quality associated with lower dementia risk in women; EPA PFAS regulations (2024)
Individual—lifestyle neuroprotection	Mediterranean/MIND diet; regular aerobic exercise; cognitive and social engagement; adequate sleep; hearing and vision correction; vascular risk factor control (BP, glucose, lipids)	Builds cognitive and physical reserve; upregulates BDNF; reduces neuroinflammation; addresses 13 of 14 Lancet modifiable risk factors	FINGER trial (Ngandu et al. 2015); Livingston et al. (2024) Lancet Commission
Clinical—exposure screening	Incorporate occupational and residential exposure history in cognitive assessment; test blood Pb, urinary Cd, serum PFAS in at-risk older adults; integrate blood AD biomarkers (p-tau181, p-tau217, Aβ42/40, GFAP)	Identifies high-risk individuals for early preventive intervention; allows monitoring of pre-clinical AD in pollution-exposed populations	Park et al. (2024); Parums (2024); Schindler et al. (2024)
Clinical—multidomain intervention	Structured programs targeting diet, exercise, cognitive training, vascular risk monitoring, and social engagement simultaneously; extend to include pollutant exposure reduction guidance	Multidomain approach more effective than single-domain for cognitive protection; addresses biological and environmental vulnerability simultaneously	FINGER trial (Ngandu et al. 2015); Livingston et al. (2024)
Policy—air quality standards	Tighten PM2.5, PM0.1, NO2 regulatory limits toward WHO 2021 guidelines (PM2.5 annual mean ≤ 5 µg/m³); enforce emission controls on transport, industry, and power generation; monitor ultrafine particles	Estimated 17% reduction in dementia risk per 10 µg/m³ PM2.5 reduction; China Clean Air Act linked to cognitive improvements in older adults	Best Rogowski et al. (2025); Calderón-Garcidueñas (2025 b); Yao et al. (2022)
Policy—chemical regulation	Accelerate phase-out of neurotoxic pesticides (chlorpyrifos, legacy OCs); enforce PFAS drinking water limits; remove lead from water infrastructure; reduce Cd in fertilizers and industrial emissions; restrict single-use plastics	Directly reduces body burden of well-documented AD-relevant neurotoxicants; regulatory feasibility demonstrated by PFAS regulations and pesticide bans in several countries	Ahmed et al. (2025) on metals; Gardener et al. (2025) on PFAS; Torres-Sánchez et al. (2023) on pesticides
Policy—environmental justice	Prioritize pollution reduction in communities of color and low socioeconomic status; equitable access to preventive healthcare; environmental impact assessment requirements for industrial siting	Disproportionate pollution exposure accounts for racial/ethnic disparities in AD incidence; lower-income countries bear greatest combined risk factor burden	Josey et al. (2023); Livingston et al. (2024); Alzheimer’s Association Facts and Figures (2024)

#### Individual-level strategies

At the individual level, several modifiable behaviors can reduce neurotoxicant exposure. The use of HEPA-filter air purifiers indoors has been demonstrated to reduce personal PM2.5 exposure, particularly in urban environments. Choosing walking routes away from high-traffic roads and exercising during low-pollution periods reduces inhaled PM2.5 and NO2 (Allen and Barn 2020). Dietary choices can significantly modify heavy metal, pesticide, and PFAS exposure: choosing organic produce reduces pesticide residue consumption, consuming low-mercury fish (sardines, anchovies, mackerel) over high-mercury species, using activated carbon-filtered tap water or tested bottled water to reduce exposure to lead, nitrates, and PFAS, and avoiding food heated in non-stick PFAS-containing cookware (González et al. 2023; Jiang et al. 2024; Souza and Domingo 2025).

Beyond exposure reduction, a suite of neuroprotective lifestyle behaviors addresses the Lancet Commission’s 14 modifiable risk factors and builds biological resilience against pollutant neurotoxicity (Depypere et al. 2025; Livingston et al. 2024). Adherence to the Mediterranean or MIND diet provides neuroprotective antioxidants, polyphenols, and omega-3 fatty acids that may partially counteract pollutant-induced oxidative stress and neuroinflammation. Regular aerobic and resistance exercise upregulates BDNF, promotes hippocampal neurogenesis, reduces neuroinflammatory markers, and improves cerebrovascular health (Romero Garavito et al. 2025; Zhou et al. 2026). Lifelong cognitive engagement and social participation build cognitive reserve, a biological buffer that reduces the clinical expression of AD pathology even in the presence of considerable neuropathological burden. Adequate sleep supports glymphatic clearance of Aβ from the brain. Treating hearing and vision loss removes sensory deprivation triggers of cognitive reserve depletion. Controlling hypertension, diabetes, obesity, and dyslipidemia addresses vascular contributors to AD risk (Livingston et al. 2024).

#### Clinical strategies

Healthcare providers can play a pivotal role by incorporating detailed occupational and residential environmental exposure histories into cognitive risk assessments of older adults (Yao et al. 2022). Identifying individuals with histories of occupational pesticide exposure, proximity to industrial point sources, or residence in high-pollution urban environments allows targeted biomarker screening. Blood Pb levels, urinary Cd, and serum PFAS panels are commercially available and can guide targeted risk reduction counseling and more intensive monitoring.

New blood-based AD biomarkers (plasma p-tau181, p-tau217, Aβ42/40 ratio, and GFAP) allow earlier, less invasive identification of preclinical AD pathology (Schindler et al. 2024). Integrating environmental exposure data with blood biomarker profiles could substantially improve risk stratification and identify the subset of pollution-exposed individuals at highest imminent risk of progression to clinical AD. Multidomain lifestyle intervention programs, exemplified by the FINGER trial (Ngandu et al. 2015), demonstrated that simultaneously targeting diet, exercise, cognitive training, and vascular risk management could significantly attenuate cognitive decline in at-risk older adults. Adapting such programs to include environmental exposure reduction counseling represents a logical and evidence-supported extension.

#### Population and policy-level strategies

The most powerful leverage points for reducing pollution-related AD risk lie at the population level. Stricter and more aggressively enforced air quality standards represent the highest-priority policy intervention given the strength of epidemiological evidence. The 2025 Lancet Planetary Health meta-analysis found that reducing PM2.5 concentrations to WHO-recommended levels (annual mean ≤ 5 µg/m³) could substantially reduce dementia incidence, particularly in Asian and South Asian megacities where ambient PM2.5 regularly exceeds 50–100 µg/m³ (Best Rogowski et al. 2025). Routine measurement and regulation of ultrafine particles, currently absent from most national monitoring networks, is urgently needed given the strong evidence from the MMC cohorts.

The phase-out of neurotoxic pesticide classes, particularly chlorpyrifos, other OPs acting on AChE, and legacy organochlorines, through regulatory action analogous to successful international bans (e.g., the Stockholm Convention on POPs) is both scientifically justified and technically achievable. Integrated pest management (IPM) and biological pest control represent viable agricultural alternatives. Accelerating the implementation of PFAS drinking water regulations across national and international jurisdictions, and restricting PFAS use in food contact materials and consumer products, can meaningfully reduce population PFAS body burden. Remediating lead in aging water infrastructure and housing stock, a persistent public health challenge in many high-income countries, and reducing cadmium in phosphate fertilizers are essential steps for heavy metal exposure reduction.

Perhaps the most important overarching policy principle is environmental justice. Environmental pollution exposure is socially and racially patterned, with communities of lower socioeconomic status, communities of color, and populations in low- and middle-income countries bearing disproportionately high exposures (Josey et al. 2023). Because these same communities face the highest AD burden and the least access to preventive healthcare, pollution reduction policies that address structural inequities will have the greatest population-level impact on AD prevention while simultaneously advancing equity. As the Lancet Commission emphasized, a higher educational level and cognitive stimulation throughout the lifespan act as bulwarks against AD (Livingston et al. 2024), and environmental justice frameworks recognize that educational and healthcare access are themselves shaped by exposure to environmental hazards.

## Limitations

Several important limitations must be acknowledged in synthesizing the evidence presented in this review. First, most epidemiological studies on pollution and AD are observational in design and are subject to residual confounding, exposure measurement error, and survivor bias. The most heavily exposed individuals may die before clinically manifesting AD, potentially underestimating true effect sizes. Second, most mechanistic data derive from animal models, or in vitro cell culture systems, which may not fully recapitulate human neurobiological complexity, while species differences in brain anatomy, metabolism, lifespan, and immunology limit direct translational extrapolation. Third, the existing scientific literature mainly examines individual pollutants in isolation, whereas real-world human exposure involves simultaneous, sequential, and synergistic exposures to complex mixtures across the entire lifespan (Domingo 2026). Mixture toxicology methods capable of capturing non-additive interactions among pollutants, such as Bayesian kernel machine regression (BKMR) and weighted quantile sum (WQS) regression, are available but underutilized in the AD literature. Fourth, the long latency period between relevant exposures (particularly those occurring in early life) and clinical AD onset makes causal inference especially challenging in epidemiological studies of finite duration, and few prospective cohorts have adequate follow-up or exposure characterization across the entire life course. Fifth, biomarker-based early diagnosis of preclinical AD is not yet universally standardized or accessible across geographic and socioeconomic contexts, limiting the ability to study environmental exposure effects in relation to early neuropathological stages rather than clinical diagnosis. Sixth, for certain emerging pollutants (PFAS and MNPs), the epidemiological evidence base for AD-specific associations remains sparse and methodologically heterogeneous, and the analytical techniques for quantifying these substances in biological matrices are still being validated and standardized.

Finally, this paper is a narrative rather than systematic review and may be subject to selection bias in the literature presented. Future systematic reviews, meta-analyses, and multidisciplinary expert panels on individual pollutant categories in relation to AD outcomes are needed to provide higher-level evidence syntheses and clearer risk quantification for regulatory and clinical purposes.

## Conclusions

Alzheimer’s disease represents one of the defining public health challenges of the 21st century. The evidence reviewed here demonstrates that this challenge cannot be adequately confronted without recognizing the critical role of environmental pollution as a modifiable driver of neurodegeneration. Ambient air pollution (PM2.5, PM0.1, and combustion-derived nanoparticles, heavy metals, pesticides, PFAS, and micro/nanoplastics) collectively constitute a neurotoxic exposome whose impact begins *in utero*, is measurable in children as young as 11 months in polluted urban environments, and accumulates over a lifetime to hasten the onset of clinical AD in biologically vulnerable individuals. At the same time, it is important to recognize that the current evidence does not support a uniform causal role for all pollutant classes discussed. The strength of evidence varies considerably, with the most robust support available for air pollution, and more limited or emerging evidence for other contaminants. Future advances will depend on the integration of longitudinal epidemiological studies, standardized exposure assessment, and validated biomarkers of early neurodegeneration.

The studies conducted by Calderón-Garcidueñas and co-workers raise the possibility that, in heavily polluted urban environments, early neuropathological changes consistent with AD features may already be present in young adults and possibly in children. This is a finding that, if confirmed through independent replication, would have major implications for when and where AD prevention efforts should be directed. At minimum, it supports the position that prevention should not be deferred to older age groups alone, and that reducing pollution exposure in childhood and early adulthood might yield long-term neuroprotective benefits. Prevention must begin in the first years of life and must be embedded in broader environmental and social justice frameworks that address the inequitable distribution of pollution burdens.

The 2024 Lancet Commission’s explicit recognition of air pollution as one of 14 modifiable risk factors for dementia, with approximately 45% of global cases potentially preventable through risk factor modification, provides both a scientific mandate and a policy framework for action. Environmental pollution reduction strategies, operating at the population level through air quality regulation, toxic chemical governance, and environmental remediation, offer the highest-leverage, most equitable, and most durable preventive interventions available, requiring no individual behavioral change and benefiting the entire exposed population simultaneously.

Future research must prioritize: (1) large-scale prospective cohort studies with multi-pollutant exposure assessment integrated with validated AD biomarker endpoints, (2) mechanistic research examining the interactions between pollution mixtures and APOE4 and other genetic susceptibility factors, (3) epidemiological studies of pollution effects on pediatric and young adult AD biomarker trajectories, (4) mixture toxicology approaches capable of quantifying synergistic neurotoxic effects, and (5) intervention studies testing whether specific pollution exposure reductions translate into measurable improvements in cognitive trajectories or biomarker profiles.

The convergence of a rapidly aging global population, persistent severe environmental pollution across many regions, and the lack of effective curative therapies for AD make its prevention an increasingly urgent and essential priority. Science, clinical medicine, and public policy must converge with equal urgency to reduce the neurotoxic burden that a polluted world places on aging brains, and on the developing brains of children who should not yet be on the trajectory toward AD. Addressing these challenges will require coordinated efforts across scientific disciplines, clinical practice, and public policy to reduce environmental exposures and better understand their contribution to neurodegenerative disease risk across the lifespan.

## Data Availability

Not applicable.
